# Contrast-enhanced MRI improves the diagnostic performance for vaginal fornix invasion in cervical carcinoma

**DOI:** 10.1186/s13244-026-02326-0

**Published:** 2026-06-04

**Authors:** Kailan Yang, Fangfang Rong, Funing Chu, Yafeng Dong, Hongkai Zhang, Bixiao Wan, Mingzhe Xu, Qingxin Xia, Lin Lu, Ho-Young Song, Hongmin Chen, Jinrong Qu

**Affiliations:** 1 Department of Radiology, The Affiliated Cancer Hospital of Zhengzhou University & Henan Cancer Hospital, Zhengzhou, China; 2 HNHC Key Laboratory of Oncology Medical Imaging Response Assessment, Henan Cancer Hospital, Zhengzhou, China; 3 Department of Gynecologic Oncology, The Affiliated Cancer Hospital of Zhengzhou University & Henan Cancer Hospital, Zhengzhou, China; 4 Department of Pathology, The Affiliated Cancer Hospital of Zhengzhou University & Henan Cancer Hospital, Zhengzhou, China; 5 Department of Medical Imaging, The Third Affiliated Hospital of Zhengzhou University, Zhengzhou, China; 6 Department of Interventional Radiology, The Affiliated Cancer Hospital of Zhengzhou University & Henan Cancer Hospital, Zhengzhou, China

**Keywords:** Uterine cervical neoplasms, Carcinoma, Vagina, Magnetic resonance imaging, Diagnostic imaging

## Abstract

**Objective:**

To investigate the added diagnostic value of contrast-enhanced MRI (CE-MRI) to T2-weighted imaging (T2WI) and diffusion-weighted imaging (DWI) for evaluating vaginal fornix invasion (VFI) in cervical carcinoma (CC).

**Materials and methods:**

We retrospectively enrolled 149 consecutive CC patients (training cohort) and 37 external patients (validation cohort) who underwent radical hysterectomy and preoperative MRI (T2WI, DWI, and CE-MRI). Two radiologists independently assessed VFI first using T2WI + DWI, then T2WI + DWI + CE-MRI, with histopathology as reference. Diagnostic metrics were compared with the McNemar test; interobserver agreement was evaluated via the kappa statistic. Subgroup analyses and multivariate logistic regression were performed to validate CE-MRI’s added value.

**Results:**

Histopathology confirmed VFI in 68/149 patients (45.6%) in the training cohort and 6/37 (16.2%) in the external validation cohort. CE-MRI clearly visualized vaginal fornix mucosal continuity and the tumor-fornix boundary, improving lesion differentiation. Interobserver agreement improved significantly from good (κ = 0.778) to excellent (κ = 0.917) with CE-MRI (Δκ = 0.139, 95% CI: 0.062–0.220, *p* = 0.003). CE-MRI significantly improved diagnostic accuracy and specificity for both observers (all *p* < 0.001), while high sensitivity remained unchanged (*p* > 0.05). Multivariate regression confirmed CE-MRI as an independent positive factor for accurate VFI diagnosis (OR = 4.05, 95% CI: 2.34–6.98, *p* < 0.001). Subgroup analysis demonstrated accuracy improvements across most clinical subgroups (all *p* < 0.05). Consistent results were obtained in the external validation cohort, with diagnostic accuracy significantly improved by CE-MRI (*p* < 0.001).

**Conclusion:**

Adding CE-MRI to T2WI + DWI significantly improves the diagnostic performance for VFI in CC.

**Critical relevance statement:**

Combining CE-MRI with T2WI + DWI significantly improves diagnostic accuracy for VFI while enhancing interobserver agreement, which will minimize miss-staging of early-stage CC, optimize treatment planning, and ultimately improve patient outcomes by reducing recurrence risk and preserving fertility when appropriate.

**Key Points:**

- Accurate assessment of vaginal fornix invasion is critical but remains radiologically challenging.
- Contrast-enhanced MRI facilitates assessing vaginal fornix mucosal continuity and superficial invasion.
- Combining contrast-enhanced MRI with T2WI + DWI enhances staging accuracy for cervical carcinoma management.

**Graphical Abstract:**

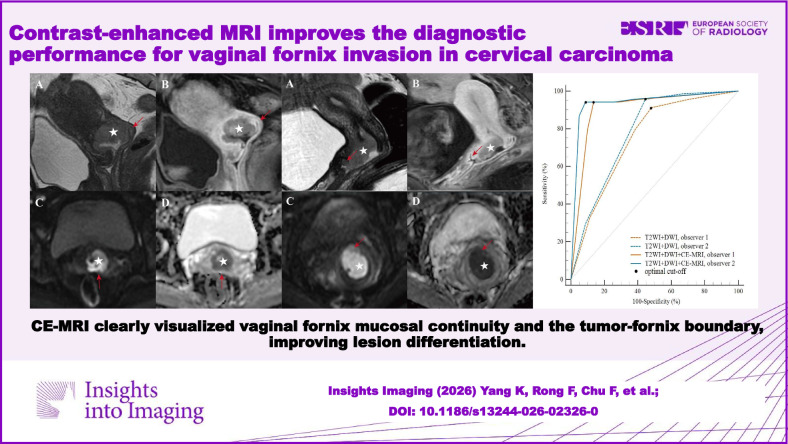

## Introduction

Cervical carcinoma (CC) ranks fourth in both incidence and mortality among women worldwide [1]. With widespread cytologic screening, CC is increasingly diagnosed in younger women and at an earlier stage; consequently, many patients are of reproductive age and may wish to preserve their fertility [2]. According to the National Comprehensive Cancer Network (NCCN) guidelines, fertility-sparing surgeries (e.g., cervical conization or radical trachelectomy) may be considered for eligible CC patients with stages IA1, IA2, or IB1 [3, 4]. In the 2018 revised International Federation of Gynecology and Obstetrics (FIGO) staging system, vaginal invasion is pivotal for early-stage CC staging: stage IB1 indicates carcinoma confined to the cervix without vaginal invasion [5]. Moreover, inaccurate vaginal invasion assessment often leads to positive or close vaginal margins after surgery, increasing recurrence risk and requiring adjuvant chemoradiotherapy [6]. As the most common invasion site at the cervicovaginal junction, accurate preoperative assessment of vaginal fornix invasion (VFI) is critical for staging, treatment planning, and prognosis in CC, but it remains challenging due to its unique anatomical characteristics.

MRI is the most reliable imaging modality for the local-regional staging of CC [7–9]. The European Society of Urogenital Radiology (ESUR) guidelines recommend T2-weighted imaging (T2WI) and diffusion-weighted imaging (DWI) as routine sequences for initial CC staging, whereas contrast-enhanced MRI (CE-MRI) remains optional [10]. T2WI without fat suppression (FS) is the mainstay, offering excellent soft-tissue contrast for detailed anatomical delineation, but it cannot reliably distinguish T2-hyperintense tumors from peritumoral edema. DWI helps differentiate tumor tissue from reactive changes but is limited in the precise evaluation of tumor margins against adjacent normal tissues due to its inherently low spatial resolution and poor anatomic detail [11]. Overestimation of VFI often occurs when bulky exophytic cervical tumors stretch the vaginal fornix or when recent biopsy-related vaginal wall edema mimics tumor involvement [12]. Although several small-scale studies have shown that intravaginal gel improves VFI assessment accuracy (with one prospective study of 28 patients reporting that the correct staging rate improved from 50% to 78.5% after vaginal opacification) [13–15], its clinical utility and benefits remain underexplored. Akita et al reported that CE-MRI outperformed T2WI in CC tumor localization, margin detection, and conspicuity [16], suggesting its potential utility for assessing VFI. Two prospective studies using T2WI and CE-MRI at 1.5 T reported accuracies of 81% (*n* = 115) and 83% (*n* = 41) for vaginal invasion [17, 18]. Crucially, these studies omitted DWI (a key sequence for CC staging) and lacked comparisons with routine protocols (T2WI + DWI), limiting their clinical relevance.

Given that the ESUR guidelines recommend T2WI and DWI as routine sequences, while the value of additional CE-MRI for VFI remains undefined, our study aimed to compare the diagnostic accuracy of VFI assessment using T2WI + DWI versus T2WI + DWI + CE-MRI.

## Materials and methods

### Study patients

This retrospective observational study was approved by the institutional review board, and the requirement for patient informed consent was waived. We screened consecutive CC patients who underwent radical hysterectomy at our hospital from August 2023 to July 2024 according to the following inclusion and exclusion criteria:

Inclusion criteria: (1) patients who underwent pelvic MRI examination at our hospital within 15 days before radical hysterectomy; (2) availability of complete MRI sequences, including T2WI, DWI, and CE-MRI; (3) complete clinical and histopathological data.

Exclusion criteria: (1) patients who underwent neoadjuvant therapy before surgery; (2) histopathology confirming FIGO stage IA1 or IA2 disease (stage IA represents microinvasive disease that is not visible at MRI [9]); (3) insufficient image quality due to severe artifacts.

**Fig. 1:**
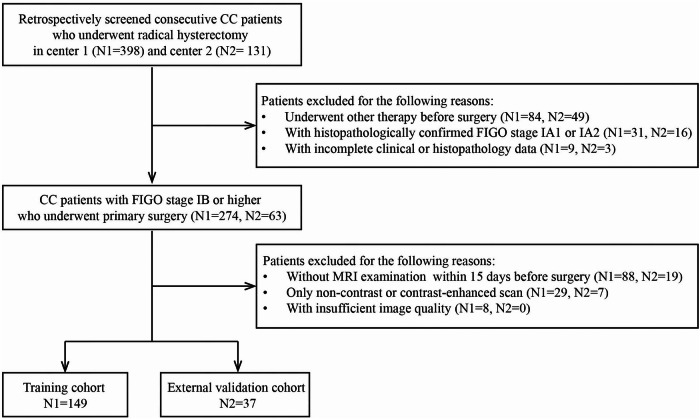
Flowchart of the study patients. CC, cervical carcinoma; CE-MRI, contrast-enhanced MRI; FIGO, International Federation of Gynecology and Obstetrics

To minimize selection bias and strengthen the robustness of our conclusions, we additionally enrolled an independent external validation cohort from the Third Affiliated Hospital of Zhengzhou University between January and August 2025, using identical inclusion and exclusion criteria. Details of the study flowchart are shown in Fig. 1.

### MR protocol

All imaging data were derived from routine clinical practice without any study-specific modification to the diagnostic protocol. All MRI examinations were performed on 3-T scanners (Supplementary Table 1) using a phased-array body coil, with multiple sequences including T2WI, DWI, and CE-MRI. Patient preparation involved fasting for at least 4 h, administering intravenous 1 mg glucagon before imaging to reduce bowel peristalsis, and achieving moderate bladder filling by partially voiding 45–60 min prior to imaging.

Oblique axial, sagittal, and coronal T2-weighted fast spin-echo sequences without FS were obtained orthogonal or parallel to the cervical canal. Using a single-shot spin-echo echo-planar imaging (SS-EPI) sequence with free breathing, DWI was acquired with b-values of 0 and 1000 s/mm^2^, and images were acquired in a plane perpendicular to the cervical canal, anatomically aligned with the oblique axial T2WI section for consistent anatomical reference. Apparent diffusion coefficient (ADC) maps were subsequently generated using the system software. CE-MRI was acquired using a three-dimensional T1-weighted gradient-echo sequence, after intravenous administration of 0.1 mmol/kg of body weight gadopentetate dimeglumine at a rate of 2.0 mL/s. The exact MRI sequence parameters are summarized in Supplementary Table 1.

### Image quality assessment

MR image quality was evaluated independently by two experienced radiologists (Y.K., observer 1, and Q.J., observer 2, each with more than 5 years of experience in pelvic MRI), who were blinded to all clinical data and histopathologic results. Using a 5-point scale, all sequences (T2WI, DWI, and CE-MRI) were scored separately as follows: 1 = poor, with prominent artifacts significantly affecting interpretation; 2 = acceptable, with moderate artifacts potentially affecting the evaluation of the cervix and vaginal fornix; 3 = average, with slight artifacts that do not affect the evaluation of the cervix and vaginal fornix; 4 = good, with clear visualization of the cervix and vaginal fornix and no significant artifacts; 5 = excellent, demonstrating excellent clarity of the cervix and vaginal fornix with no artifacts. In cases of inter-rater discrepancy, the lower score was adopted as the final score. According to the Standards for Reporting Diagnostic Accuracy Studies (STARD) criteria, images with a score of 1 were classified as unsatisfactory and excluded from subsequent analyses [19].

### MR assessment of VFI

All MR images were reviewed independently by the two radiologists. Furthermore, L.L. (observer 3, 9 years of experience in pelvic MRI) from the Third Affiliated Hospital of Zhengzhou University independently assessed the external validation cohort to ensure reproducibility of the findings, particularly given the small sample. To minimize recall bias, at least a 1-month interval was maintained between interpretation session 1 (T2WI + DWI) and interpretation session 2 (T2WI + DWI + CE-MRI).

CC typically manifests as intermediate-to-hyperintense lesions on T2WI, with high-signal on high-b-value DWI and corresponding low-signal on ADC maps, exhibiting early arterial enhancement similar to the mucosa and becoming isointense or hypointense relative to the stroma in the delayed phase [2]. The normal vaginal fornix wall exhibits a three-layered pattern: the inner mucosa is T2-hyperintensity; the middle muscularis propria is T2-hypointensity; and the outer adventitia is T2-hyperintensity [20]. All these layers exhibit no diffusion restriction on DWI and show homogeneous enhancement on CE-MRI.

**Fig. 2:**
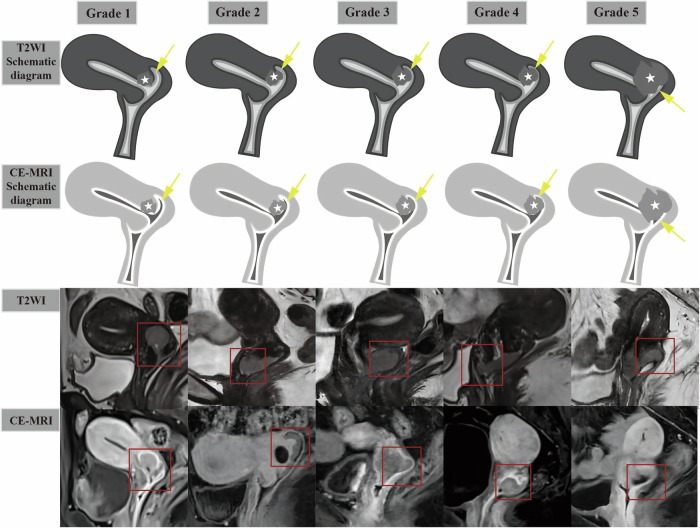
Schematic diagram and typical MR images characteristics of the 5-point grading scale for vaginal fornix invasion (VFI) in cervical carcinoma (CC) on T2WI and CE-MRI. Yellow arrow: vaginal fornix mucosa; White star: cervical carcinoma tumor

Diagnostic criteria for VFI were stratified by different imaging protocols: for T2WI + DWI alone, interruption of the hypointense vaginal fornix wall (compared to intermediate-to-hyperintense tumor) on T2WI, accompanied by corresponding restricted diffusion on DWI; for T2WI + DWI + CE-MRI, the aforementioned T2WI + DWI findings, plus abnormal enhancement extending from the tumor to the vaginal fornix wall on CE-MRI [9]. To distinguish false-positive findings associated with the T2 shine-through effect, each interpretation of DWI was combined with the ADC map. To determine the presence or absence of VFI, a 5-point grading scale was used: 1 = definitely absent; 2 = probably absent; 3 = indeterminate; 4 = probably present; and 5 = definitely present (Table 1 and Fig. 2). Grades 4 and 5 were regarded as positive for VFI, whereas grades 1 to 2 were regarded as negative; grade 3 was further stratified as positive or negative based on the optimal cutoff value determined by receiver operating characteristic (ROC) curve analysis. Postoperative histopathologic results served as the reference standard. All specimens were processed using standardized protocols. Diagnoses were established by pathologists with > 5 years of experience and verified by a senior pathologist to ensure accuracy.

**Table 1:** 5-point grading scale for vaginal fornix invasion (VFI)

Grade	Definition
1	Clear, smooth, and continuous vaginal fornix wall on all sequences; no tumor in proximity
2	Tumor in close proximity to vaginal fornix, but the fornix wall remains continuous (sometimes separated by a thin T2-hyperintense and/or non-enhancing mucus signal layer)
3	Ambiguous continuity of T2-hyperintense and/or hyper-enhanced vaginal fornix mucosa due to tumor compression (no definite disruption)
4	Tumor tightly attached to the vaginal fornix wall, with blurred fornix mucosa
5	Definite tumor-induced interruption of vaginal fornix wall continuity (involving the mucosa, muscularis propria, and/or adventitia)

### Statistical analysis

Statistical analyses were performed by using Medcalc (version 23.4.8) and R (version 4.3.1), and a *p* < 0.05 was considered statistically significant.

### Diagnostic performance

Diagnostic efficiencies of T2WI + DWI and T2WI + DWI + CE-MRI were compared using ROC curves and the Delong test; the optimal cutoff values were determined by Youden’s index. Sensitivity, specificity, positive predictive value (PPV), negative predictive value (NPV), and accuracy were calculated and compared using the McNemar test.

### Interobserver agreement

Interobserver agreement was evaluated using linear weighted kappa test, with statistical comparisons performed using the Bootstrap method (1000 iterations). Kappa values were interpreted as follows: < 0.20, poor agreement; 0.21–0.40, fair agreement; 0.41–0.60, moderate agreement; 0.61–0.80, good agreement; and > 0.80, excellent agreement.

### Subgroup analysis

Based on tumor size (≤ 4 cm vs. > 4 cm), menopausal status (premenopausal vs. postmenopausal), FIGO stage (early (IB1-IIA) vs. advanced (≥ IIB)), and pathological type (squamous cell carcinoma vs. non-squamous cell carcinoma), a comprehensive subgroup analysis was conducted. Diagnostic accuracies were compared within each subgroup using the McNemar test. Interaction testing was performed using logistic regression to evaluate whether the benefit of CE‑MRI varied across subgroups.

### Multivariable analysis

A multivariate logistic regression model (generalized estimating equations, GEE) was constructed, with accurate VFI diagnosis as the outcome variable and CE-MRI application, tumor size, menopausal status, FIGO stage, and histologic type as independent variables to adjust for confounders.

### Supplementary analyses

The Mann–Whitney U test was used to compare image quality scores between correctly and incorrectly diagnosed cases to assess whether image quality confounded diagnostic accuracy. A post hoc power analysis for the McNemar test was conducted with an alpha level of 0.05.

### External validation

The optimal VFI diagnostic cutoff values derived from the primary training cohort were applied to the external validation cohort. AUCs and diagnostic performance metrics were compared between the two protocols using the DeLong and McNemar tests, respectively.

## Results

### Image quality and diagnostic impact

In the training cohort, 149 cases were initially included, and 8 with a score of 1 were excluded from subsequent analyses; all 37 cases in the external validation had a score ≥ 2. The inter-rater agreement in assessing T2WI, DWI, and CE-MRI image quality was excellent (all κ > 0.80) (Table 2). Comparison of image quality scores between correctly and incorrectly diagnosed patients revealed no consistent confounding effect on diagnostic accuracy (Supplementary Table 2). For observer 1, no significant differences were detected in any sequence or protocol (all *p* > 0.05). For observer 2, only a single significant difference was found in T2WI under the T2WI + DWI protocol (*p* = 0.023), which was not reproduced in other sequences or the combined protocol.

**Table 2:** MR image quality assessment by observers 1 and 2

		Score 1	Score 2	Score 3	Score 4	Score 5	Kappa
Training cohort (*n* = 157)							
T2WI	Observer 1	2	3	12	45	95	0.866 (0.810–0.922)
	Observer 2	2	4	11	42	98	
DWI	Observer 1	6	5	34	72	40	0.923 (0.880–0.965)
	Observer 2	8	3	33	76	37	
CE-MRI	Observer 1	1	3	10	31	112	0.887 (0.831–0.944)
	Observer 2	1	4	9	25	118	
External validation cohort (*n* = 37)							
T2WI	Observer 1	0	0	3	17	17	0.820 (0.662–0.978)
	Observer 2	0	0	1	19	17	
DWI	Observer 1	0	1	7	26	3	0.843 (0.680–0.994)
	Observer 2	0	0	9	25	3	
CE-MRI	Observer 1	0	0	5	21	11	0.826 (0.666–0.985)
	Observer 2	0	0	4	23	10	

### Patient characteristics

A total of 149 patients in the training cohort and 37 in the external validation cohort were finally enrolled, with patient characteristics summarized in Table 3. No statistically significant differences in baseline characteristics between the included patients and the 117 patients excluded for missing or incomplete MRI sequences (all *p* > 0.05), eliminating potential selection bias from baseline differences (Supplementary Table 3).

**Table 3:** Patient characteristics

Characteristics	Training cohort	External validation cohort
No. of patients	149	37
Age (years)	53.97 ± 12.33	52.78 ± 10.96
HPV infection		
Positive	139	35
Negative	10	2
Menstrual status		
Premenopausal	57	13
Postmenopausal	92	24
Histoathologically confirmed VFI	68 (45.6%)	6 (16.2%)
Tumor size (cm)	3.90 ± 1.56	3.29 ± 0.91
FIGO stage		
IB1	12	16
IB2	43	10
IB3	16	1
IIA1	39	6
IIA2	16	0
IIIC1p	22	4
IIIC2p	1	0
Histologic type		
Squamous cell carcinoma	122	29
Adenocarcinoma	22	8
Neuroendocrine carcinoma	4	0
Adenoid cystic carcinoma	1	0

### Diagnostic performance in the training cohort

Histopathology confirmed VFI in 68 of 149 patients (45.6%). Using the optimal cutoff values (grade > 2 for T2WI + DWI, > 3 for T2WI + DWI + CE-MRI), observer 1 diagnosed VFI as positive in 101 (67.8%) and 75 (50.3%) patients for the two protocols, respectively; observer 2 made positive diagnoses in 101 (67.8%) and 71 (47.7%) patients, respectively.

Interobserver agreement for VFI was good with T2WI + DWI (κ = 0.778, 95% CI: 0.705–0.852) and excellent with T2WI + DWI + CE-MRI (κ = 0.917, 95% CI: 0.875–0.958); the difference in kappa values was statistically significant (Δκ = 0.139, 95% CI: 0.062–0.220, two-tailed *p* = 0.003).

**Fig. 3:**
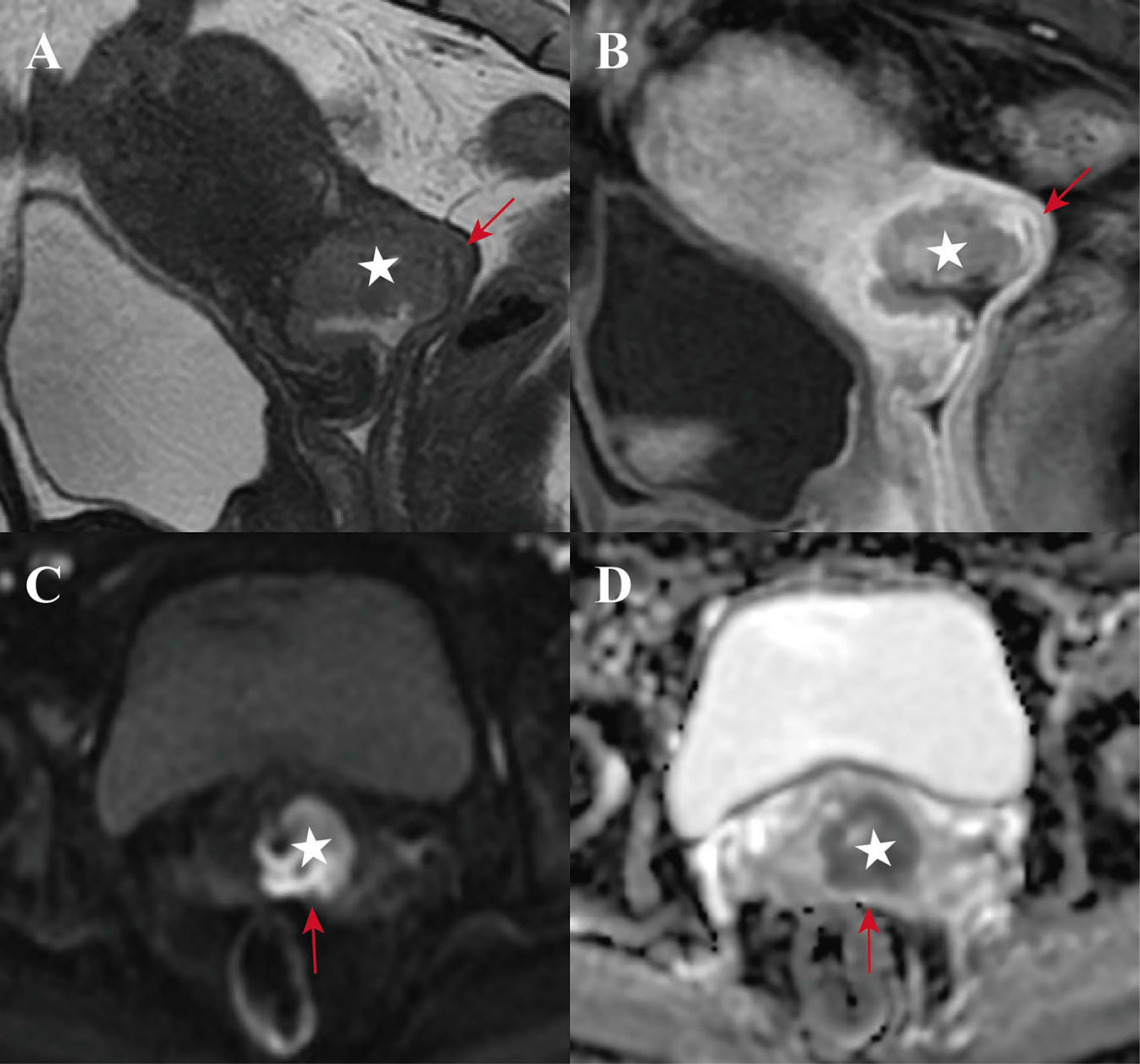
Representative images in a 47-year-old woman with stage IB2 cervical squamous cell carcinoma. Oblique sagittal T2-weighted image (**A**) shows a slightly hyperintense cervical tumor (star), with an irregular margin and disruption of the hypointense posterior vaginal fornix wall (red arrow). Diffusion-weighted image (**C**) and apparent diffusion coefficient map (**D**) show that the peritumoral edema causes a diagnostic pitfall. Contrast-enhanced T1-weighted image (**B**) demonstrates the tumor margin more distinctly and a continuous mucosa of the posterior vaginal fornix. Grades 3 and 2 were assigned by observer 1 and 2, respectively, when interpreting T2WI + DWI, but grade 1 was assigned by both observers when interpreting T2WI + DWI + CE-MRI. No evidence of invasion into the vaginal fornix was confirmed by histopathology

**Fig. 4:**
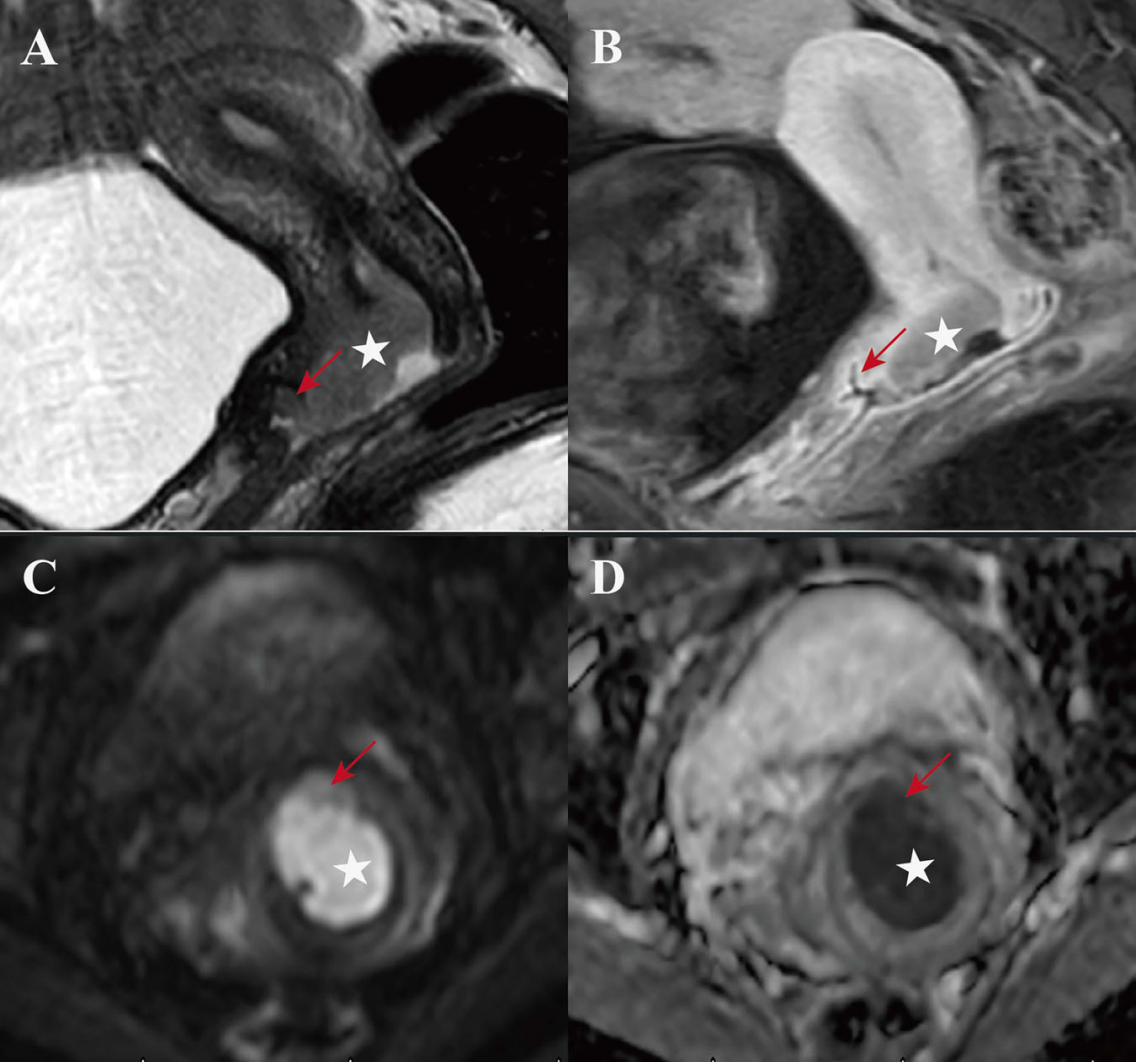
Representative images in a 30-year-old woman with stage IB2 cervical squamous cell carcinoma. Oblique sagittal T2-weighted image (**A**) shows a slightly hyperintense cervical tumor (star) abutting the anterior vaginal fornix wall (red arrow). Combined analysis of diffusion-weighted image (**C**) and apparent diffusion coefficient map (**D**) reveals disruption of the vaginal fornix wall. However, contrast-enhanced T1-weighted image (**B**) demonstrates that the tumor is at a certain distance from the anterior vaginal fornix wall. Grade 5 was assigned by both observers when interpreting T2WI + DWI, but grade 1 was assigned by both observers when interpreting T2WI + DWI + CE-MRI. No invasion of the vaginal fornix was confirmed by histopathology

**Fig. 5:**
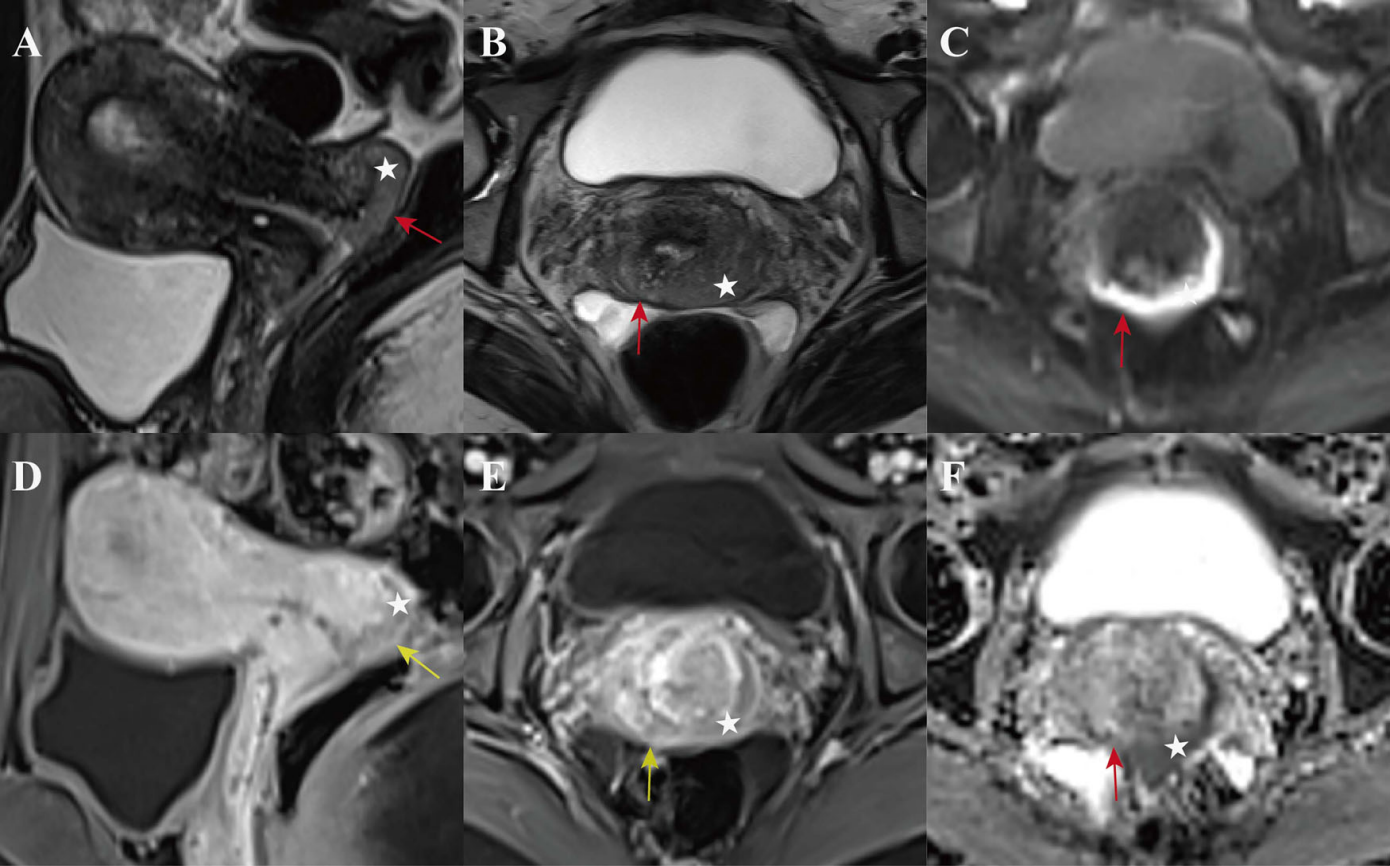
Representative images in a 31-year-old woman with stage IIA1 cervical squamous cell carcinoma. Oblique sagittal T2-weighted image (**A**), oblique axial T2-weighted image (**B**), diffusion-weighted image (**C**), and apparent diffusion coefficient map (**F**) show an exophytic cervical tumor (star) tightly stretching the posterior vaginal fornix, thereby making the wall too thin to assess its continuity (red arrow). Sagittal (**D**) and axial (**E**) contrast-enhanced T1-weighted images demonstrate an irregular vaginal fornix wall with serrated changes (yellow arrow). Grade 3 was assigned by both observers when interpreting T2WI + DWI, but grade 4 was assigned by both observers when interpreting T2WI + DWI + CE-MRI. Vaginal fornix invasion was confirmed by histopathology

The McNemar test showed high baseline sensitivity of VFI diagnosis was preserved after adding CE-MRI (both observers, *p* > 0.05), whereas specificity and overall accuracy were significantly improved (all *p* < 0.001) (Table 4). PPV and NPV also increased with the combined protocol. Representative cases are illustrated in Figs. 3–5. Post hoc power analysis showed a power of > 0.999 (exceeding the 80% threshold), confirming an adequate sample size.

**Table 4:** Comparisons between T2WI + DWI and T2WI + DWI + CE-MRI for the diagnostic performance of VFI in CC (training cohort)

Parameters	T2WI + DWI		T2WI + DWI + CE-MRI		*p*-value^a^	
	Observer 1	Observer 2	Observer 1	Observer 2	Observer 1	Observer 2
Sensitivity (%)	91.2 (81.8–96.7)	95.6 (87.6–99.1)	94.1 (85.6–98.4)	94.1 (85.6–98.4)	0.688	1.000
Specificity (%)	51.9 (40.5–63.1)	55.6 (44.1–66.6)	86.4 (77.0–93.0)	91.4 (83.0–96.5)	< 0.001	< 0.001
Positive predictive value (%)	61.4 (55.6–66.8)	64.4 (58.5–69.8)	85.3 (77.0–91.0)	90.1 (81.8–94.9)	-	-
Negative predictive value (%)	87.5 (76.0–93.9)	93.8 (83.0–97.9)	94.6 (87.1–97.8)	94.9 (87.7–98.0)	-	-
Accuracy (%)	69.8 (61.7–77.0)	73.8 (66.0–80.7)	89.9 (83.9–94.3)	92.6 (87.2–96.3)	< 0.001	< 0.001
Kappa^b^	0.778 (0.705–0.852)		0.917 (0.875–0.958)		-	-

Data in parentheses are 95% confidence intervals

^a^ McNemar test

^b^ Weighted Kappa test (linear weights)

**Fig. 6:**
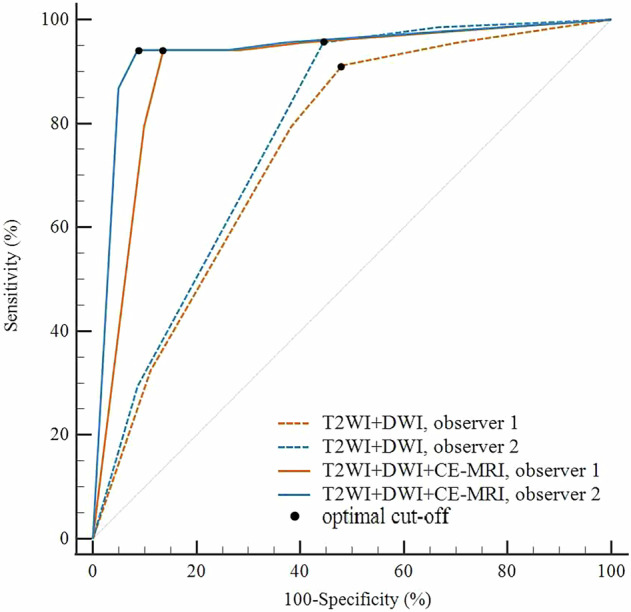
ROC curves for vaginal fornix invasion (VFI) assessment using T2WI + DWI and T2WI + DWI + CE-MRI. Optimal cutoff values were defined by the Youden Index. For observer 1, the AUC increased significantly from 0.751 (95% CI, 0.674–0.818) for T2WI + DWI (optimal cutoff > 2) to 0.907 (95% CI, 0.849–0.949) for T2WI + DWI + CE-MRI (optimal cutoff > 3) (*p* < 0.001). For observer 2, the AUC exhibited a similar significant increase from 0.783 (95% CI, 0.708–0.846) for T2WI + DWI (optimal cutoff > 2) to 0.939 (95% CI, 0.887–0.971) for T2WI + DWI + CE-MRI (optimal cutoff > 3) (*p* < 0.001)

The addition of CR-MRI significantly increased AUC values: observer 1’s AUC increased from 0.751 (95% CI: 0.674–0.818) to 0.907 (95% CI: 0.849–0.949) and observer 2’s increased from 0.783 (95% CI: 0.708–0.846) to 0.939 (95% CI: 0.887–0.971), both with *p* < 0.001 (Fig. 6).

### Subgroup analysis and multivariable analysis (training cohort)

T2WI + DWI + CE-MRI consistently outperformed T2WI + DWI alone across most subgroups, including tumor size ≤ 4 cm and > 4 cm, premenopausal and postmenopausal status, early FIGO stage (IB-IIA), and squamous cell carcinoma (all *p* < 0.05) (Supplementary Table 4). Improvements did not reach statistical significance in patients with advanced-stage disease (*n* = 23*, p* = 0.375) or non-squamous cell carcinoma (*n* = 27, *p* = 0.250). FIGO stage was the only stratification with a significant interaction (*p* = 0.011).

Multivariable analysis confirmed that CE-MRI was an independent positive factor for accurate VFI diagnosis (OR = 4.05, 95% CI: 2.34–6.98, *p* < 0.001) after adjusting for tumor size (*p* = 0.886), menopausal status (*p* = 0.943), or FIGO stage (*p* = 0.418). Pathological type was a minor independent influencing factor (OR = 0.17, *p* = 0.006) but did not alter the strong independent diagnostic value of CE-MRI (Supplementary Table 5).

### External validation cohort

When the cutoff values from the training cohort were applied to the independent external validation cohort (*n* = 37), results were highly consistent with the primary analysis (Table 5). Adding CE‑MRI significantly improved overall accuracy from 45.9% to 89.9% for observer 1 and from 51.4% to 91.9% for observer 3 (both *p* < 0.001). Specificity was also markedly improved (both *p* < 0.001), while high sensitivity was maintained.

**Table 5:** Diagnostic performance in the external validation cohort (*n* = 37)

Parameters	T2WI + DWI		T2WI + DWI + CE-MRI		*p*-value^a^	
	Observer 1	Observer 3	Observer 1	Observer 3	Observer 1	Observer 3
Sensitivity (%)	100 (54.1–100)	100 (54.1–100)	100 (54.1–100)	100 (54.1–100)	-	-
Specificity (%)	35.5 (19.2–54.6)	41.9 (24.5–60.9)	80.6 (62.5–92.5)	90.3 (74.2–97.9)	< 0.001	< 0.001
PPV (%)	23.1 (18.9–28.0)	25.0 (19.8–31.0)	50.0 (32.8–67.2)	66.7 (40.6–85.4)	-	-
NPV (%)	100 (100–100)	100 (100–100)	100 (100–100)	100 (100–100)	-	-
Accuracy (%)	45.9 (29.5–63.1)	51.4 (34.4–68.1)	89.9 (83.9–94.3)	91.9 (78.1–98.3)	< 0.001	< 0.001
AUC	0.820 (0.659–0.926)	0.879 (0.730–0.963)	0.968 (0.850–0.999)	0.976 (0.863–0.999)	0.050	0.165
Kappa^b^	0.792 (0.670–0.913)		0.902 (0.827–0.977)		-	-

Metrics were calculated using the optimal cutoff values derived from the training cohort (grade > 2 for T2WI + DWI and > 3 for T2WI + DWI + CE-MRI). Data in parentheses are 95% confidence intervals

^a^ McNemar test (sensitivity, specificity, and accuracy); Delong test (AUC)

^b^ Weighted Kappa test (linear weights)

## Discussion

Our retrospective study demonstrated that adding CE-MRI to the standard T2WI + DWI protocol significantly improves diagnostic performance for preoperative VFI assessment in CC. This combined approach improved diagnostic accuracy from approximately 70% to nearly 90% for both observers and also achieved excellent interobserver agreement, highlighting its considerable clinical value in preoperative staging.

Gynecological examination remains the primary clinical method for evaluating VFI, but it relies on visual evaluation and is frequently confounded by mild erosion, congestion, or inflammatory changes [21]. Canaz et al reported that clinical staging tends to overestimate vaginal invasion compared with histopathology [22]. A large multicenter retrospective study of 23,933 CC patients identified vaginal invasion as the leading cause of staging inaccuracy (62.3%), with 72.4% and 70.9% of stage IIA1 and IIA2 patients, respectively, clinically overstaged [23]. Inaccurate staging directly impacts oncologic outcomes: up to 8% of stage IB CC patients undergoing radical surgery had positive or close vaginal margins, increasing recurrence risk [24].

Since the 2018 revised FIGO staging system endorsed cross-sectional imaging, MRI has become the most reliable imaging modality for local-regional staging of CC. Nevertheless, studies specifically focusing on VFI evaluation remain limited. T2WI without FS can effectively demonstrate tumor and normal cervical/vaginal fornix anatomy, especially on the sagittal plane. When combined with DWI, it helps distinguish some false-positive findings caused by peritumoral edema. However, its performance is imperfect. In our study, 3.0-T T2WI + DWI achieved PPV values of 61.4% and 64.4% for observers 1 and 2, respectively; a substantial number of false positives remained (39 and 36 cases, respectively). This can be attributed to several factors: first, bulky exophytic tumors can compress and stretch the fornix, rendering assessment of its integrity difficult [25]; second, the inherently low spatial resolution of DWI limits precise evaluation of the tumor margin relative to adjacent normal tissue; and third, in postmenopausal women, the vaginal fornix often becomes shallow or obliterated, complicating the delineation of normal anatomical structures [26].

Several recent studies have highlighted the superiority of CE-MRI in the assessment of CC tumors. Akita et al reported that CE-MRI provided clearer delineation of tumor margins compared to T2WI, with both sequences achieving 95% accuracy in assessing infiltration of the vagina [16]. However, this study lacked DWI, enrolled only 40 patients (38 without vaginal invasion), and did not compare against a routine T2WI + DWI protocol, limiting direct clinical extrapolation. Our study directly compared the routine ESUR-recommended protocol (T2WI + DWI) against an enhanced protocol (T2WI + DWI + CE-MRI) in a larger cohort (*n* = 149 training, *n* = 37 validation). We quantitatively demonstrated that CE-MRI addition significantly increased the AUC (0.751–0.783 to 0.907–0.939), specificity (51.9–55.6% to 86.4–91.4%), and overall accuracy (~ 70% to ~ 90%), while high sensitivity was retained (> 91.2%). Compared with prior studies, Choi et al and Sheu et al reported accuracies of 81% (*n* = 115) and 83% (*n* = 41) for vaginal invasion diagnosis using 1.5-T T2WI + CE-MRI without DWI, with a low PPV of 51% and a high overstaging rate (6/30 non-invasive cases), respectively [17, 18]. In contrast, our 3.0-T T2WI + DWI + CE-MRI protocol yielded a PPV of 85.3–90.1%, which was markedly higher than the 51% of prior T2WI + CE-MRI studies and the 61.4–64.4% of T2WI + DWI in our cohort. Multivariate analysis further confirmed CE-MRI as an independent predictor of accurate VFI diagnosis after adjusting for multiple clinicopathological factors. To our knowledge, this is the first study to validate the added value of CE-MRI within the complete ESUR-recommended MRI protocol, providing robust and clinically applicable evidence.

In our study, hyperintense mucosa is frequently obscured on T2WI by vaginal fornix collapse and hyperintense luminal fluid, but clearly visualized on early-phase CE-MRI owing to its prominent early enhancement. CE-MRI not only depicted tumor margins more clearly but also visualized the vaginal fornix mucosa more distinctly, significantly facilitating assessment of mucosal continuity and superficial invasion. The reduction in false-positive diagnoses (from 36–39 to 7–11 cases) was mainly attributed to improved differentiation of tumor invasion from benign mimics such as fornix edema or compression by exophytic tumors. False-negative cases (3–6 with T2WI + DWI, 4 with CE-MRI) often involved minimal tumor vaginal fornix invasion, difficult to identify on imaging. Compared with T2WI + DWI, interpreting T2WI + DWI + CE-MRI significantly increased PPV and specificity for VFI.

Our findings carry distinct clinical value: this study does not simply advocate CE-MRI, but demonstrates that adding CE-MRI to the ESUR-recommended T2WI + DWI protocol improves preoperative staging accuracy in early-stage CC and translates this benefit into tangible clinical advantages. The optimized protocol significantly reduces VFI miss-staging by improving diagnostic specificity and overall accuracy, thereby supporting individualized treatment strategies. For eligible IB1 patients, reducing false-positive results avoids unnecessary radical hysterectomy and preserves fertility. Meanwhile, accurate detection of VFI prevents incomplete resection and positive vaginal margins, reduces the need for adjuvant chemoradiotherapy, lowers recurrence risk, and ultimately improves survival and quality of life.

This study has several limitations. First, as a retrospective study, selection bias due to the predefined exclusion criteria may limit the generalizability of our findings. Patients who received preoperative radiation or chemotherapy were excluded to avoid confounding effects, so our findings are most applicable to the preoperative staging of CC patients being considered for surgical management. FIGO IA1/IA2 cases were excluded because microinvasion is usually MRI-occult, and our focus was on differentiating IB (no VFI) from IIA (with VFI). Second, recall bias could potentially impact imaging interpretation. To address this, we adopted a blinded approach for assessing clinical and histopathologic outcomes and ensured an interval of at least 1 month between imaging evaluations. Third, the single-center training cohort had a moderate sample size. Although we validated our protocol in an independent external cohort with consistent results, and conducted comprehensive subgroup and multivariate analyses, larger prospective multicenter studies are warranted to confirm the added value of CE-MRI in underrepresented subgroups (e.g., advanced stage, non-squamous histology) and to establish standardized CE-MRI interpretation criteria for VFI.

In conclusion, the addition of CE-MRI to the standard T2WI + DWI protocol significantly improves diagnostic performance, interobserver agreement, and reader confidence for assessing VFI in patients with cervical carcinoma, offering a reliable means to refine staging and guide treatment.

## Supplementary information

ELECTRONIC SUPPLEMENTARY MATERIAL

## Data Availability

The original data supporting the findings of this study are readily available upon request. They can be obtained from the corresponding author without any undue delay.

## Abbreviations

ADC: Apparent diffusion coefficient
AUC: Area under the curve
CC: Cervical carcinoma
CE-MRI: Contrast-enhanced magnetic resonance imaging
DWI: Diffusion-weighted imaging
ESUR: European Society of Urogenital Radiology
FIGO: International Federation of Gynecology and Obstetrics
FS: Fat suppression
NCCN: National Comprehensive Cancer Network
NPV: Negative predictive value
PPV: Positive predictive value
ROC: Receiver operating characteristic
STARD: Standards for Reporting Diagnostic Accuracy Studies
T2WI: T2-weighted imaging
VFI: Vaginal fornix invasion
