# Supplementary material for: Contrast-enhanced MRI improves the diagnostic performance for vaginal fornix invasion in cervical carcinoma
Source: Insights Imaging. 2026 Jun 4;17:152. doi: 10.1186/s13244-026-02326-0 (PMC13237291; doi:10.1186/s13244-026-02326-0)
Supplement: Supplementary file 1 — ELECTRONIC SUPPLEMENTARY MATERIAL [file 13244_2026_2326_MOESM1_ESM.pdf]

Contrast-enhanced MRI improves the diagnostic performance for vaginal fornix invasion in cervical carcinoma

ELECTRONIC SUPPLEMENTARY MATERIAL

Insights Imaging (2026) Yang K, Rong F, Chu F, et al.

Supplementary Table 1 Detailed MRI sequence parameters

Center	3-T scanners	Parameters	T2WI Oblique axial	T2WI Oblique sagittal	T2WI Oblique coronal	DWI (0 and 1000 s/mm^2)	CE-MRI Axial	CE-MRI Sagittal	CE-MRI Coronal
Center 1 (training cohort)	Philips Ingenia CX	TR/TE (ms)	3000/120	3200/100	3100/100	3600/57	5.1/1.97	4.3/1.56	4.2/1.53
		Matrix	320×320	288×286	312×320	100×136	300×360	300×332	356×348
		Voxel (mm×mm×mm)	0.75×0.75×4	0.8×0.8×3	0.8×0.96×3	2.21×2.46×5	1×1×5	0.9×1×4	0.9×1×4
		FOV (mm×mm)	240×240	230×230	260×300	245×300	300×360	300×300	320×340
		Slice thickness (mm)	4	3	3	5	2.5	2	2
		Gap (mm)	0.4	0.3	0.3	0.5	0	0	0
		NEX	1	1	1	8	1	1	1
	UIH uMR 770	TR/TE (ms)	4200/130	3900/130	3900/130	2800/71	5.07/2.37	4.39/2.04	4.34/2
		Matrix	336×336	336×336	320×320	100×176	352×352	306×304	336×336
		Voxel (mm×mm×mm)	0.71×0.71×4	0.83×0.83×4	0.81×0.81×4	2.05×2.05×4	1.02×1.02×5	0.92×0.92×4	0.95×0.95×4

Center	3-T scanners	Parameters	T2WI Oblique axial	T2WI Oblique sagittal	T2WI Oblique coronal	DWI (0 and 1000 s/mm^2)	CE-MRI Axial	CE-MRI Sagittal	CE-MRI Coronal
		FOV (mm×mm)	240×240	280×280	260×260	260×360	260×360	280×280	320×320
		Slice thickness (mm)	4	4	4	4	2.5	2	2
		Gap (mm)	0.4	0.4	0.4	0.4	0	0	0
		NEX	2	1	1.1	4	1	1	1
	GE	TR/TE (ms)	4200/120	3400/102	5500/102	4800/77	6.2/1.27	8.3/2.5	5.8/2.5
	SIGNA	Matrix	352×352	304×304	288×288	168×142	352×384	288×320	320×320
	Architect	Voxel (mm×mm×mm)	0.7×0.7×4	0.9×0.9×3	0.9×0.9×3	2.1×2.5×5	1.1×1×4.8	1×1.1×4.8	1.1×1.1×5
		FOV (mm×mm)	240×240	260×260	260×260	360×360	304×380	310×310	350×350
		Slice thickness (mm)	4	3	3	5	2.5	2.5	2.5
		Gap (mm)	0.4	1	1	0.5	0	0	0
		NEX	2	1	1	4	1	1	1
	SIEMEN	TR/TE (ms)	11000/120	4600/120	4800/120	7100/65	3.41/1.29	3.99/1.36	3.48/1.27
	S	Matrix	300×384	358×448	288×384	108×150	208×352	240×320	348×320

Center	3-T scanners	Parameters	T2WI Oblique axial	T2WI Oblique sagittal	T2WI Oblique coronal	DWI (0 and 1000 s/mm^2)	CE-MRI Axial	CE-MRI Sagittal	CE-MRI Coronal
	Prisma	Voxel (mm×mm×mm)	0.8×0.63×4	0.73×0.58×4	0.9×0.68×4	2.93×2.93×5	1.49×1.19×5	1.25×1×4	1.64×1.31×4.8
		FOV (mm×mm)	240×240	260×260	260×260	316×420	310×420	240×320	407×420
		Slice thickness (mm)	4	4	4	5	2.5	2	2.4
		Gap (mm)	0.4	0.4	0.4	0.5	0	0	0
		NEX	2	1	1	8	1	1	1
	SIEMENS	TR/TE (ms)	11000/120	4600/120	4800/120	7100/65	3.41/1.29	3.99/1.36	3.48/1.27
		Matrix	300×384	358×448	288×384	108×150	208×352	240×320	348×320
	Skyra	Voxel (mm×mm×mm)	0.8×0.63×4	0.73×0.58×4	0.9×0.68×4	2.93×2.93×5	1.49×1.19×5	1.25×1×4	1.64×1.31×4.8
		FOV (mm×mm)	240×240	260×260	260×260	316×420	310×420	240×320	407×420
		Slice thickness (mm)	4	4	4	5	2.5	2	2.4
		Gap (mm)	0.4	0.4	0.4	0.5	0	0	0

Center	3-T scanners	Parameters	T2WI Oblique axial	T2WI Oblique sagittal	T2WI Oblique coronal	DWI (0 and 1000 s/mm^2)	CE-MRI Axial	CE-MRI Sagittal	CE-MRI Coronal
		NEX	2	1	1	8	1	1	1
Center 2 (external validation cohort)	Philips Ingenia	TR/TE (ms)	3500/85	3137/110	3299/90	2368/64	5.1/1.97	4.3/1.56	4.2/1.53
		Matrix	320×320	389×284	231×320	87×92	222×268	252×252	251×292
		FOV (mm×mm)	240×240	256×267	256×266	245×300	400×418	352×367	352×367
		Slice thickness (mm)	4	5	4	5	2.5	2	2
		Gap (mm)	0.4	0.4	0.4	0.5	0	0	0
		NEX	2	1	1	8	1	1	1
	GE SIGNA Pioneer	TR/TE (ms)	4200/120	6300/81	4260/80	4000/79	4.5/1.88	3.9/1.72	3.98/1.89
		Matrix	352×352	288×288	244×320	128×128	360×360	240×272	192×288
		FOV (mm×mm)	240×240	240×250	280×290	300×310	360×376	300×310	300×310
		Slice thickness (mm)	5	4	5	5	2	3	3
		Gap (mm)	0.4	1	1	0.5	0	0	0
		NEX	2	1	1	6	1	1	1

Center	3-T scanners	Parameters	T2WI Oblique axial	T2WI Oblique sagittal	T2WI Oblique coronal	DWI (0 and 1000 s/mm^2)	CE-MRI Axial	CE-MRI Sagittal	CE-MRI Coronal
	SIEMENS Skyra	TR/TE (ms)	3500/85	3500/85	3500/85	5100/51	3.31/1.30	3.71/1.75	3.85/1.74
		Matrix	288×384	320×320	288×384	95×120	195×320	320×320	216×288
		FOV (mm×mm)	280×380	320×320	288×384	380×380	380×380	380×396	400×417
		Slice thickness (mm)	4	5	5	4	3	1.6	2
		Gap (mm)	0.8	0.8	0.8	1	0	0	0
		NEX	1	1	1	4	1	1	1

Supplementary Table 2 Comparison of MRI image quality scores between correctly and incorrectly diagnosed patients (Mann-Whitney U test)

Observer(method)	Sequence	Image quality scores(mean) Correctly diagnosed patients	Incorrectly diagnosed patients	U value	*p* value
Observer 1 (T2WI+DWI)	T2WI	4.62	4.42	1948	0.057
	DWI	3.99	3.89	2186	0.489
Observer 1 (T2WI+DWI+CE-MRI)	T2WI	4.58	4.40	837	0.213
	DWI	3.96	3.93	1002	0.984
	CE-MRI	4.70	4.73	998.5	0.957
Observer 2 (T2WI+DWI)	T2WI	4.63	4.36	1697	0.023*
	DWI	3.95	3.97	2121.5	0.912
Observer 2 (T2WI+DWI+CE-MRI)	T2WI	4.52	4.36	619	0.233
	DWI	4.00	3.90	752	0.956
	CE-MRI	4.66	4.82	697.5	0.553

* Indicates statistical significance (two-sided Mann-Whitney U test, $p < 0.05$)

Supplementary Table 3 Baseline characteristics of included patients versus those excluded for missing or incomplete MRI

Characteristics	Included patients	Excluded patients	p^a value
No. patients	149	117	
Age (years)	53.97±12.33	52.32±9.82	0.228
Menstrual status			0.549
premenopausal	57	49	
postmenopausal	92	68	
Tumor size (cm)	3.90±1.56	3.92±1.68	0.986
FIGO stage			0.122
IB1	12	7	
IB2	43	46	
IB3	16	21	
IIA1	39	18	
IIA2	16	9	
IIIC1p	22	15	
IIIC2p	1	1	
Histologic type			0.434
squamous cell carcinoma	122	100	
non-squamous cell carcinoma	27	17	

[a] two-sample t-test (age and tumor size); Chi-Square test (menstrual status, FIGO stage, and histologic type).

Supplementary Table 4 Comparisons between T2WI+DWI and T2WI+DWI+CE-MRI for the diagnostic performance of VFI in CC in different subgroups (Observer 1)

Subgroup	n	T2WI+DWI Accuracy (%)	T2WI+DWI+CE-MRI Accuracy (%)	p^a value	interaction p^b value
Overall	149	69.8 (61.7-77.0)	89.9 (83.9-94.3)	<0.001	
Tumor size（cm）					0.768
≤4	102	70.6 (60.7-79.2)	89.2 (81.5-94.5)	0.035	
>4	47	68.1 (52.8-80.9)	91.5 (79.6-97.6)	<0.001	
Menstrual status					0.239
premenopausal	57	68.4 (54.8-80.1)	93.0 (83.0-98.1)	0.002	
postmenopausal	92	70.3 (59.8-79.5)	88.0 (79.4-93.8)	0.007	
FIGO Stage					0.011
early stage (IB-IIA)	126	69.8 (61.0-77.7)	92.9 (86.9-96.7)	<0.001	
advanced stage (≥IIB)	23	69.6 (47.1-86.8)	73.9 (51.6-89.8)	0.375	
Histologic type					0.545
squamous cell carcinoma	122	64.6 (55.6-73.2)	88.5 (81.5-93.6)	<0.001	
non-squamous cell carcinoma	27	92.6 (75.7-99.1)	96.3 (81.0-99.9)	0.250	

Note—Data in parentheses are 95% confidence intervals.

[a] McNemar test. [b] Logistic Regression.

Supplementary Table 5 Multivariate logistic regression analysis of factors associated with accurate VFI diagnosis

Variable	Odds Ratio (OR)	95% CI	P-value
Protocol (T2WI+DWI+CE-MRI vs. T2WI+DWI)	4.05	2.34-6.98	<0.001
Tumor Size (Continuous)	1.02	0.77-1.36	0.886
Menstrual status (Postmenopausal vs. Premenopausal)	0.97	0.46-2.06	0.943
FIGO Stage (IIICp vs. IB-IIA)	0.68	0.27-1.73	0.418
Histologic type (squamous cell carcinoma vs. others)	0.17	0.05-0.61	0.006

Note—The model was fitted using Generalized Estimating Equations (GEE) to account for repeated measures within patients.
